# Accuracy of Veterans Affairs Databases for Diagnoses of Chronic Diseases

**Published:** 2009-09-15

**Authors:** Jasvinder A. Singh

**Affiliations:** Minneapolis VA Medical Center. Dr Singh is affiliated with the University of Minnesota, Minneapolis, Minnesota, and the Mayo Clinic School of Medicine, Rochester, Minnesota

## Abstract

**Introduction:**

Epidemiologic studies usually use database diagnoses or patient self-report to identify disease cohorts, but no previous research has examined the extent to which self-report of chronic disease agrees with database diagnoses in a Veterans Affairs (VA) health care setting.

**Methods:**

All veterans who had a medical care visit from October 1, 1996, through May 31, 1998, at any of the Veterans Integrated Service Network 13 facilities were surveyed about physician diagnosis of chronic obstructive pulmonary disease (COPD)/asthma, depression, diabetes, and heart disease. Four administrative case definitions (data from VA databases) consisting of combinations of International Classification of Diseases, Ninth Revision, codes and disease-specific medication data were compared with self-report of each disease to assess sensitivity, specificity, positive and negative predictive values, area under receiver operating characteristics curve, and κ statistic.

**Results:**

Sensitivity for administrative definitions compared with self-report of physician diagnosis was 24% to 54% for COPD/asthma, 25% to 47% for depression, 27% to 59% for heart disease, and 64% to 78% for diabetes. Specificity was 88% to 100% for all diseases. The κ statistic showed fair agreement for COPD/asthma, depression, and heart disease and substantial agreement for diabetes.

**Conclusion:**

Diagnoses identified from databases agree with self-report for diabetes but not COPD/asthma, depression, or heart disease in a VA health care setting.

## Introduction

Large epidemiologic or health services studies usually resort to using administrative or clinical databases (eg, Medicare, Medicaid, Veterans Affairs, health maintenance organizations) or patient self-report (eg, data from the Behavioral Risk Factor Surveillance System) for case detection. Few studies, however, have examined the extent to which self-reported diagnoses agree with those obtained from databases. Self-reported diagnoses have good agreement with those obtained from databases for hypertension (1) and diabetes (2), but self-reported (3-8) and database-derived (9,10) data have variable rates of agreement with diagnoses obtained from medical records review and physical examination. Two studies that combined diagnoses from databases with prescription information found that the combination was more accurate than either method alone for hypertension (1) and diabetes (11).

The Veterans Health Administration is the largest integrated health care system in the United States. One Veterans Affairs (VA) study compared diagnostic accuracy in veterans with serious mental illness and found that they are less aware of comorbidities (12), but to my knowledge, no previous study has examined the extent to which self-report of chronic diseases agrees with diagnoses from databases in a VA health care setting. Veterans are sicker and have more comorbidites than do age-matched Americans in general (13), and since comorbidity is associated with less accuracy of self-report (4), rates of agreement may be lower and predictors different in a veteran cohort than in the general population. In the general population, younger age, better cognition, more education, less comorbidity, female sex, being married, and more frequent use of medical services are associated with more accurate self-report (4,5,7,14-16). Alternatively, a small social network, major depression, recent alcohol abuse, and serious mental illness are associated with less accurate self-report (6,12).

In a study of quality of life of veterans receiving health care in Veterans Integrated Service Network 13 (VISN-13), data were collected regarding patient self-report of chronic diseases, administrative diagnoses, and use of medications (17). These data were used to examine the extent to which patient self-report of physician diagnosis agrees with data obtained from administrative databases and whether patient demographic, clinical, or functional parameters affect the agreement.

## Methods

### Veterans' Quality of Life Study

The Veterans' Quality of Life Study was a cohort study of all veterans who received inpatient or outpatient health care at any of the VISN-13 facilities (covering all of Minnesota, North Dakota, and South Dakota and selected counties in Iowa, Nebraska, Wisconsin, and Wyoming) from October 1, 1996, through May 31, 1998, and had a valid mailing address (17). This cohort of veterans was mailed a survey, and a repeat mailing was sent to nonresponders. The survey response rate was 58% (40,508 of 70,334 eligible veterans).

The survey included questions regarding 1) self-report of physician diagnosis of chronic conditions, including chronic obstructive pulmonary disease (COPD) or asthma, depression, diabetes, heart disease, hypertension, and arthritis; 2) demographic information, including sex, education level, and race/ethnicity; 3) smoking status; and 4) functional limitation as assessed by limitation of activities of daily living, such as bathing, dressing, eating, getting in and out of a chair, walking, and using the bathroom (18). The survey also included the SF-36V (Short Form Health Survey for Veterans) (19), which consists of 8 subscales: physical functioning, bodily pain, general health, vitality, mental health, social functioning, role emotional, and role physical (role limitations due to emotional or physical problems, respectively). Physical and mental component summary scores of SF-36V were generated from the 8 subscales, standardized to the US population, and norm-based; possible values ranged from 0 to 100, and higher scores corresponded to better health.

Prospective and retrospective cohort data were obtained for the year before and the year after the survey from the Patient Treatment File and the Outpatient Clinic data sets in the VA administrative databases at the Austin Automation Center in Austin, Texas. These data are reliable for demographic characteristics and most common diagnoses (20) and valid for specific diagnoses (9,10). Data extracted included demographics (age, marital status, employment status, and percentage service connection) and health care use. A veteran is considered "service connected" if he or she has disabilities resulting from or beginning during active military duty, and veterans with a service connection of 50% or higher get priority access to VA care. Data regarding number of inpatient hospitalizations and outpatient visits in primary care, specialty medical care, surgical care, and mental health were extracted and categorized according to stop codes.

### Validation study

In addition to the above data, for this study International Classification of Diseases, Ninth Revision (ICD-9), codes and prescription data for 4 self-reported comorbidities (COPD/asthma, depression, diabetes, and heart disease), and Current Procedural Terminology codes for percutaneous transluminal coronary angioplasty and coronary artery bypass grafting only for heart disease, were extracted from each facility for the 2-year period, including the year before and the year after the survey (Table 1). The pharmacy database (VISN-13 Veterans Health Information Systems and Technology Architecture) was searched for 2 or more refills of the prescriptions specific to each condition that were available in the VA pharmacy during the 2-year period of this search. Only disease-specific medications were searched rather than all medications, since this strategy was intended to be specific (and not sensitive) for case detection. From the pharmacy and ICD-9 code information, the following 4 database case definitions were formulated for each disease and compared with self-report of physician diagnosis: ICD-9 code, medication use, ICD code or medication use, and ICD-9 code and medication use.

### Statistical analyses

The accuracy of various administrative database case definitions was calculated for each disease by comparing them with patient self-report of physician diagnosis for each condition. The agreement between database case definitions and self-report was assessed by calculating the κ statistic (21). The measures of accuracy included sensitivity, specificity, positive and negative predictive values, and area under the receiver operating characteristics curve. Sensitivity analyses were performed by considering the 4 administrative case definitions for the year before the survey or the 2-year period including 1 year before and 1 year after the survey. Sensitivity was defined as the fraction of patients who reported physician diagnosis of a condition that was correctly identified as positive for that condition by each administrative database case definition (ICD only, medication only, ICD or medication, ICD and medication). Specificity was defined as the fraction of those who did not report diagnosis of a condition that was correctly identified as negative for the condition by each database case definition. Positive (or negative) predictive values were the fraction of cases with positive (or negative) data definitions (those with both self-reported physician diagnosis and the case definition or with neither) among all patients with (or without) data definition. Results are presented for the definitions that included the 1-year period (before the survey), since they did not differ substantially from those that included the 2-year period.

Multivariable logistic regression analyses were performed to determine the factors significantly associated with disagreement between self-report and administrative database definition for various chronic diseases. To avoid multiple analyses, the database case definition of ICD-9 code in the year before the survey was used. This definition was chosen for multiple reasons: 1) ICD-9 code is frequently used for case detection in large epidemiologic studies, 2) ICD-9 code is easy to extract from most large databases, and 3) this administrative case definition was associated with the most agreement (highest κ statistic) with the self-report case definition in most instances. The year before the survey was chosen for the definition because patients can report a disease only if they were told of the diagnosis by their physician before the survey (and not after the survey). Various predictor factors that were modeled in these regression analyses included demographics; clinical measures; health care use, access, and eligibility measures; and health and functional status. For the purpose of analysis, outpatient visits, physical component summary, and mental component summary scores were divided into tertiles. All participants were included in the main logistic regression analysis, and differences were considered significant at *P* < .05.

## Results

Participants with various conditions had similar outpatient and inpatient use (Table 2). More patients with depression reported at least 1 mental health visit, service connection, and unemployment than did patients with other conditions. Smoking was more prevalent in patients with COPD/asthma and depression than in patients with diabetes or heart disease.

Fair-to-moderate agreement for COPD/asthma, depression, and heart disease and substantial agreement for diabetes were found (Table 3). In general, κ statistics were the highest when the most inclusive administrative case definition was examined (either ICD-9 code or medication use) and lowest when the strictest definition was considered (both ICD-9 code and medication use). κ statistics were similar when administrative data definitions during a 2-year period were considered rather than the prior year (data not shown).

Sensitivity and negative predictive value were highest for the administrative case definition of ICD-9 code or medication use, and specificity and positive predictive value were highest for the administrative case definition of ICD-9 code and medication use (Table 3). For example, for diabetes, the sensitivity and positive predictive values were, respectively, 76% and 91% for the ICD-9 code definition (most sensitive definition), which implies that 76% of patients who reported physician-diagnosed diabetes had an ICD-9 code for it, and 91% of those with an ICD-9 code for diabetes could correctly identify their diagnosis. Similarly, the specificity and positive predictive values of 100% and 98% for ICD-9 code and medication use definition for diabetes (most specific definition) indicates that all patients who reported absence of physician-diagnosed diabetes lacked an ICD-9 code and diabetes medication prescription for it and that 98% of those who did not have an ICD-9 code or diabetes medication prescription could correctly identify the absence of a diabetes diagnosis. Results were similar when administrative data definitions during a 2-year period were considered instead of the 1-year period (data not shown).

Lower number of outpatient visits, higher number of comorbidities, and lower physical component summary score were associated with higher odds of disagreement for most chronic diseases (Table 4). Some factors had opposite effects on disagreement in different diseases; older age, for example, was associated with 10% to 20% lower odds of disagreement for diagnosis of depression but 90% to 190% higher odds of disagreement for diagnosis of heart disease. Sex was not associated with disagreement between self-report and administrative database definitions for any disease.

## Discussion

This study of elderly veterans in VISN-13 found fair-to-moderate agreement between administrative definitions and self-report of COPD/asthma, heart disease, and depression and substantial agreement for diabetes. High κ and positive predictive values for administrative database definitions versus self-report for diabetes confirm similar earlier findings of κ 0.70 to 0.93 (3,22-28) and positive predictive value of 77% to 94% (3,23,29). The present study extends these findings to VA databases. This study differs slightly from previous studies in terms of comparison of self-report to databases in this study versus medical chart documentation (3,22,24) or physical examination findings (23). The study most similar in design to this one (2) found κ of 0.81 between Medicaid claims data and self-reported diabetes in a sample of 2,154 adult Medicaid recipients in Oregon (2).

The finding of a much higher level of agreement between self-report and administrative database diagnosis of diabetes as compared with COPD/asthma, depression, and heart disease confirms similar previous findings in heart disease (3,23,28), COPD (28), and depression (23). It also supports the assertion that if a disease is conceptually clear (for example, diabetes), severe, or persistent, it is easily communicated by the doctor to the patient (23). In addition, ambiguity of some survey questions, differences in patient knowledge and perception by disease, and specificity of medications for particular diseases may have contributed to differences in level of agreement. For example, the question regarding heart disease asked patients about "myocardial infarction, heart attack, or heart problems including angina," which may not be as easily understood by patients as the question about diabetes.

That higher number of comorbidities and older age increased discrepancy (decreased κ) between self-report and database diagnoses confirmed an earlier finding of lower κ between self-report and medical records–based algorithms in women aged 65 years or older (4), in a random sample of Olmsted County residents (30), and in a representative sample of Finnish residents aged 45 to 73 years (3) and is in contrast to findings in a study of patients with end-stage renal disease (28). The studies differ in that self-report was compared with database diagnoses in this study and with medical records-based algorithms (4) or physician diagnosis and medical record (3,28,30) in the other studies.

Findings of more disagreement in nonwhite and less educated patients confirm similar findings (8). Lower physical or mental health status and being nonwhite were associated with higher odds of disagreement for the chronic conditions. This finding may be secondary to increased recall bias in these groups. For COPD/asthma, being a smoker was associated with 60% higher odds of disagreement, which may be secondary to underreporting of COPD/asthma by smokers because of denial or overdocumentation of COPD/asthma diagnosis by physicians.

More physician visits are associated with more disagreement between self-report and medical record evidence for cardiovascular diseases (3). In the present study, increased outpatient use was associated with lower discordance for heart disease, which may be secondary to more effective patient-physician communication.

This study has several limitations. The nonresponse bias and cohort characteristics (elderly veterans, predominantly male and white) may limit the generalizability. However, these data should be useful to VA epidemiologists who use computerized databases. Shortcomings in the questionnaire design may also have influenced the level of agreement, as previously described (24). On the other hand, use of more specific questions (such as asking about coronary artery disease) may lead to even more disagreement because lay people may not be familiar with the vocabulary. For epidemiologic studies, neither self-report nor diagnosis from databases are standards, but they are the most common methods for identifying cohorts. The validity was examined in only 1 VISN of the VA system and may not reflect coding practices for the entire VA. Since the data are more than 10 years old, some codes or coding practices may have changed or VA data sets may be more complete or accurate now. Finally, patients who participated in this study may have been different from the general VA population, which would introduce selection bias.

This study also has several strengths. The sample was large, and results were robust across database definitions, including various combinations of ICD-9 codes and prescription of disease-specific medication. The self-report definition in this study is, in fact, self-report of physician diagnosis, which is more accurate than self-report alone.

These findings also have clinical implications. The finding that 89% to 91% of elderly veterans with COPD/asthma, depression, or heart disease who are being treated for the condition (ICD-9 code plus medication) could identify their diagnosis implies that these veterans can be identified without accessing medical records and could be targeted for interventions at a community level, such as education on self-management, healthy lifestyles, and exercise and other nonpharmacologic interventions. These interventions may be even more relevant for patients with diabetes (exercise, weight reduction, foot care, and self-monitoring), since 98% can identify their disease.

In summary, agreement between self-report of physician diagnosis and database diagnoses differs by the diagnosis. Agreement is fair to moderate for COPD/asthma, heart disease, and depression and substantial for diabetes. The effect of patient demographic, clinical, health care use, and access measures underscores the limitation of common approaches that use patient self-report or administrative databases to identify disease cohorts. Further studies should develop algorithms to improve the methods of patient cohort selection.

## Figures and Tables

**Table 1:** ICD-9 Codes and Medications Used to Determine Disease Diagnoses

**Disease**	**ICD-9 Codes^a^**	**Medications^b^**
Chronic obstructive pulmonary disease/asthma	490 (bronchitis not specified as acute or chronic), 491 (chronic bronchitis), 492 (emphysema), 493 (asthma), 495 (extrinsic allergic alveolitis), 496 (chronic airway obstruction not elsewhere classified)	Albuterol inhaler/MDI, metaproterenol inhaler/MDI, formoterol inhaler/MDI, salmeterol inhaler/MDI, beclomethasone inhaler/MDI, flunisolide inhaler/MDI, fluticasone inhaler/MDI, budesonide inhaler/MDI, ipratropium bromide inhaler/MDI, cromolyn sodium inhaler/MDI, bitolterol mesylate aerosol, isoetharine aerosol, albuterol aerosol, pirbuterol aerosol, terbutaline, terbutaline sulfate, aminophylline, dyphylline, oxtriphylline, theophylline, theophylline SR, ephedrine sulfate, montelukast, nedocromil sodium, racemic epinephrine
Depression	296.xx (affective psychoses), 300.4x (neurotic depression/dysthymic disorder), 301.1x (affective personality disorder), 298 (other nonorganic psychosis), 311 (depression disorder not elsewhere classified)	Doxepin, clomipramine, amoxapine, nortriptyline, trazodone, venlafaxine, amitriptyline, maprotiline, fluvoxamine, isocarboxazid, phenelzine, desipramine, tranylcypromine, paroxetine, fluoxetine, mirtazapine, nefazodone, trimipramine, imipramine, protriptyline, bupropion, sertraline, citalopram, escitalopram
Diabetes mellitus	250 (diabetes mellitus)	Metformin, acarbose, insulin, repaglinide, glimepiride, glyburide, chlorpropamide, glipizide, troglitazone, pioglitazone, rosiglitazone, tolazamide, tolbutamide, acetohexamide
Heart disease	410 (acute myocardial infarction), 411 (other acute and subacute forms of ischemic heart disease), 412 (old myocardial infarction), 413 (angina pectoris), 414 (other forms of chronic ischemic heart disease) **CPT codes:** 3601-3609 (percutaneous transluminal coronary angioplasty), 3610-3619, 362, 363 (coronary artery bypass grafting)	Isosorbide dinitrate, nitroglycerin, aspirin, enteric-coated aspirin

Abbreviations: ICD-9, International Classification of Diseases, Ninth Revision; MDI, metered-dose inhaler; SR, sustained release.

a For heart disease, some codes listed are Current Procedural Terminology (CPT) codes, as indicated.

b Because some medications were dispensed as brand-name drugs, proprietary names were also included in the search, but only generic names are listed.

**Table 2:** Demographic, Clinical, and Health Care Use Characteristics of Veterans With Self-Reported Chronic Diseases

Characteristic	Chronic Disease [mean (SD) or %]			

	COPD/Asthma (9,309-10,135)^a^	Depression (10,016-10,754)^a^	Heart Disease (10,761-11,676)^a^	Diabetes (6,469-7,066)^a^
**Age, y**	66 (12)	62 (14)	68 (11)	68 (11)
**Men**	96	95	98	97
**White^b^**	96	95	97	94
**Married**	65	57	70	66
**Education**				
Less than 8th grade	23	18	23	23
Some high school	13	11	13	13
High school graduate	34	34	35	34
At least some college	29	37	29	29
**Employment status**				
Employed	25	27	24	23
Unemployed	19	28	16	16
Retired	51	40	55	55
Unknown	5	6	6	5
**Current smoker**	26	31	17	18
**≥1 inpatient stay/y**	17	17	18	17
**≥1 mental health visit/y**	16	37	13	13
**No. of visits/y**				
Primary care clinic	4 (4)	3 (4)	4 (4)	4 (4)
Specialty medicine clinic	2 (4)	2 (5)	2 (5)	2 (6)
Surgery clinic	2 (3)	2 (3)	2 (3)	3 (4)
**1-year mortality**	6	5	6	6
**Multiple site use**	11	13	12	11
**Any service connection**	39	46	39	38

Abbreviations: SD, standard deviation; COPD, chronic obstructive pulmonary disease.

a Range of number of patients indicates the number of patients for whom survey responses or database information were available. The number varies by the question because some questions were skipped.

b Data on race were available for only 67% of patients.

**Table 3:** Accuracy of Administrative Case Definitions Compared With Self-Report of Chronic Diseases

Chronic Disease	% (95% CI)				ROC Area (95% CI)	κ (95% CI)
	Sensitivity	Specificity	PPV	NPV		
**COPD/asthma**						
ICD-9 code	49 (48-49)	94 (93-94)	73 (72-73)	84 (84-84)	0.71 (0.71-0.72)	0.47 (0.47-0.48)
Medication use	29 (28-29)	96 (95-96)	70 (69-70)	79 (79-80)	0.62 (0.62-0.63)	0.30 (0.30-0.31)
Either ICD-9 code or medication use	54 (53-54)	90 (90-91)	66 (65-66)	85 (84-85)	0.72 (0.71-0.73)	0.46 (0.46-0.47)
Both ICD-9 code and medication use	24 (23-24)	99 (99-99)	91 (90-91)	79 (78-79)	0.62 (0.61-0.62)	0.30 (0.30-0.31)
**Depression**						
ICD-9 code	34 (33-34)	97 (97-98)	83 (83-84)	79 (78-79)	0.65 (0.65-0.66)	0.38 (0.37-0.39)
Medication use	38 (38-39)	94 (94-94)	72 (71-72)	80 (79-80)	0.66 (0.66-0.67)	0.38 (0.38-0.39)
Either ICD-9 code or medication use	47 (46-47)	93 (92-93)	72 (71-72)	82 (81-82)	0.70 (0.69-0.70)	0.44 (0.44-0.45)
Both ICD-9 code and medication use	25 (25-26)	99 (99-99)	89 (89-89)	77 (77-78)	0.62 (0.61-0.63)	0.31 (0.30-0.32)
**Heart disease**						
ICD-9 code	36 (35-36)	97 (97-97)	86 (86-87)	75 (74-75)	0.66 (0.66-0.67)	0.38 (0.38-0.39)
Medication use	50 (49-50)	89 (89-90)	70 (70-71)	78 (77-78)	0.70 (0.69-0.70)	0.37 (0.36-0.38)
Either ICD-9 code or medication use	59 (59-60)	88 (87-88)	71 (71-72)	81 (80-81)	0.73 (0.73-0.74)	0.49 (0.48-0.50)
Both ICD-9 code and medication use	27 (26-27)	99 (98-99)	90 (90-90)	72 (72-73)	0.63 (0.62-0.63)	0.30 (0.30-0.31)
**Diabetes**						
ICD-9 code	76 (75-76)	98 (98-98)	91 (91-91)	95 (94-95)	0.87 (0.86-0.88)	0.79 (0.79-0.80)
Medication use	66 (66-67)	100 (100-100)	97 (97-98)	93 (93-93)	0.83 (0.82-0.84)	0.75 (0.75-0.76)
Either ICD-9 code or medication use	78 (78-79)	98 (98-98)	91 (91-91)	95 (95-95)	0.88 (0.88-0.89)	0.81 (0.81-0.82)
Both ICD-9 code and medication use	64 (63-64)	100 (100-100)	98 (97-98)	92 (92-93)	0.82 (0.81-0.82)	0.73 (0.73-0.74)

Abbreviations: CI, confidence interval; PPV, positive predictive value; NPV, negative predictive value; ROC, receiver operating characteristics; COPD, chronic obstructive pulmonary disease; ICD-9, International Classification of Diseases, Ninth Revision.

**Table 4:** Factors Significantly Associated With Overall Discordance Between Self-Report and ICD-9 Code for Each Chronic Disease

Predictor	Odds Ratio (95% Confidence Interval)			

	COPD/Asthma	Depression	Heart Disease	Diabetes
**Age, y**				
≤50	NS	1 [Reference]^a^	1 [Reference]^a^	1 [Reference]^a^
51-65	NS	0.9 (0.8-1.0)	1.9 (1.7-2.1)	1.4 (1.1-1.8)
>65	NS	0.8 (0.7-0.9)	2.9 (2.6-3.3)	1.9 (1.5-2.4)
**Marital status**				
Married	NS	1 [Reference]^a^	NS	NS
Unmarried	NS	1.3 (1.2-1.4)	NS	NS
**Education**				
Less than 8th grade	NS	NS	1 [Reference]^a^	NS
Some high school	NS	NS	0.9 (0.8-1.0)	NS
High school graduate	NS	NS	0.9 (0.8-0.9)	NS
At least some college	NS	NS	0.9 (0.8-1.0)	NS
**Race**				
White	NS	1 [Reference]^b^	1 [Reference]^c^	1 [Reference]^a^
Nonwhite	NS	1.1 (1.0-1.3)	1.2 (1.1-1.3)	1.5 (1.2-1.9)
**Employment status**				
Employed	NS	1 [Reference]^c^	NS	NS
Unemployed	NS	1.2 (1.1-1.3)	NS	NS
Retired	NS	1.0 (0.9-1.1)	NS	NS
**Smoking status**				
Nonsmoker	1 [Reference]^a^	NS	NS	NS
Smoker	1.6 (1.5-1.8)^a^	NS	NS	NS
**No. of comorbidities**				
0	NS	1 [Reference]^a^	1 [Reference]^a^	1 [Reference]^a^
1	NS	1.4 (1.3-1.6)	1.2 (1.1-1.3)	1.3 (1.0-1.7)
2	NS	1.6 (1.4-1.8)	1.4 (1.3-1.6)	1.7 (1.3-2.1)
≥3	NS	2.2 (2.0-2.6)	1.9 (1.7-2.1)	2.1 (1.7-2.7)
**No. of ADL limitations**				
0	1 [Reference]^a^	1 [Reference]^a^	NS	NS
1	1.3 (1.2-1.5)	0.7 (0.7-0.8)	NS	NS
2	1.6 (1.4-1.8)	0.7 (0.6-0.8)	NS	NS
≥3	2.1 (1.8-2.4)	0.9 (0.8-1.0)	NS	NS
**Prior hospitalizations**				
0	1 [Reference]^b^	NS	NS	1 [Reference]^b^
≥1	1.1 (1.0-1.2)	NS	NS	0.8 (0.6-1.0)
**Medical site use**				
Multiple site	NS	1 [Reference]^c^	NS	NS
Single site	NS	1.2 (1.1-1.4)	NS	NS
**Service connection, %**				
0	NS	1 [Reference]^a^	NS	1 [Reference]^c^
10-50	NS	1.1 (1.0-1.2)	NS	1.1 (0.9-1.3)
>50	NS	1.4 (1.2-1.5)	NS	1.4 (1.1-1.7)
**No. of outpatient visits**				
Highest tertile	1 [Reference]^b^	1 [Reference]^a^	NS	1 [Reference]^a^
Middle tertile	1.0 (0.9-1.1)	1.1 (1.0-1.1)	NS	1.2 (1.0-1.4)
Lowest tertile	1.1 (1.0-1.2)	1.4 (1.3-1.6)	NS	2.2 (1.8-2.5)
**PCS score**				
Lowest tertile	1 [Reference]^a^	NS	1 [Reference]^a^	1 [Reference]^b^
Middle tertile	0.8 (0.8-0.9)	NS	0.8 (0.7-0.9)	0.9 (0.8-1.1)
Highest tertile	0.6 (0.5-0.6)	NS	0.6 (0.5-0.6)	0.7 (0.6-0.9)
**MCS score**				
Lowest tertile	1 [Reference]^a^	1 [Reference]^a^	NS	NS
Middle tertile	1.0 (0.9-1.0)	0.4 (0.3-0.4)	NS	NS
Highest tertile	0.8 (0.7-0.9)	0.1 (0.1-0.1)	NS	NS

Abbreviations: ICD-9, International Classification of Diseases, Ninth Revision; COPD, chronic obstructive pulmonary disease; NS, not significant; ADL, activities of daily living; PCS, physical component summary; MCS, mental component summary.

a *P* < .001.

b *P* < .01.

c *P* < .05.
